# *Mycobacterium tuberculosis*: An Emerging Disease of Free-Ranging Wildlife

**DOI:** 10.3201/eid0806.010358

**Published:** 2002-06

**Authors:** Kathleen A. Alexander, Eve Pleydell, Mark C. Williams, Emily P. Lane, John F.C. Nyange, Anita L. Michel

**Affiliations:** * Centre for Conservation of African Resources: Communities Animals and Land Use, Kasane, Botswana; † University of Cambridge, Cambridge, United Kingdom; ‡ University of Pretoria, Onderstepoort, South Africa; § National Veterinary Laboratory, Gaborone, Botswana; ¶ ARC-Onderstepoort Veterinary Institute, Onderstepoort, South Africa

**Keywords:** emerging disease, tuberculosis, *Mycobacterium tuberculosis*, wildlife, sanitation, HIV

## Abstract

Expansion of ecotourism-based industries, changes in land-use practices, and escalating competition for resources have increased contact between free-ranging wildlife and humans. Although human presence in wildlife areas may provide an important economic benefit through ecotourism, exposure to human pathogens may represent a health risk for wildlife. This report is the first to document introduction of a primary human pathogen into free-ranging wildlife. We describe outbreaks of *Mycobacterium tuberculosis*, a human pathogen, in free-ranging banded mongooses *(Mungos mungo)* in Botswana and suricates *(Suricata suricatta)* in South Africa. Wildlife managers and scientists must address the potential threat that humans pose to the health of free-ranging wildlife.

Tuberculosis (TB), considered an important emerging disease in humans, is now the leading cause of death in adults worldwide (1). Although *Mycobacterium tuberculosis* is the most common infection in humans, *M. bovis* is responsible for an increasing proportion of human TB cases (1). *M. bovis* is widespread in domestic animals and has been extensively documented in both captive and free-ranging wildlife populations (2). A number of wildlife populations are endemically infected, for example, the European badger (*Meles meles*) in the United Kingdom (3) and the African buffalo (*Syncerus caffer*) in South Africa (2). These permanent reservoirs of infection pose a serious threat to public health and TB eradication programs. In contrast, *M. tuberculosis* is considered primarily a human pathogen and has been reported only in domestic or wildlife species living in close, prolonged contact with humans (4,5). We describe the first documented outbreak of *M. tuberculosis* in free-ranging wildlife and discuss the implications for ecotourism and wildlife health.

## Material and Methods

Twelve troops of free-living suricates were monitored daily by behavioral ecologists from the universities of Cambridge and Pretoria (6). The groups occupied ranges on uncultivated ranch land along the dry bed of the Kuruman River in the southern Kalahari Desert in Botswana (S 25° 8´, E 20° 49´). Complete information on outbreak features was not available. One animal was captured and euthanized for postmortem examination.

An epizootic affecting banded mongooses (*Mungos mungo*) was first identified at the northern extreme of Chobe National Park along the Chobe River in the dry season, from June 13 to September 15, 1999 (S 17° 49.33´, E 25° 07.58´). To monitor the progression of the outbreak, morning and evening patrols were conducted along the range occupied by the respective troops, and all observations, geographic features of importance, and affected animals were georeferenced**.** Surveillance was thought to be comprehensive and not biased in terms of road systems, as the patrolled roads were parallel to the river (Figure) and the animals had no other water source during the dry season. Thus, all known troops lived along the watercourse in the floodplain rather than inland in the woodland. All infected animals were euthanized whenever possible, and postmortem examinations were conducted. The rate of identification of new clinical cases from the point of outbreak, where the first case was identified, into Kasane Township and the National Park was calculated as the average time (days) between identification of each new case divided by the distance (kilometers) between cases.

**Figure:**
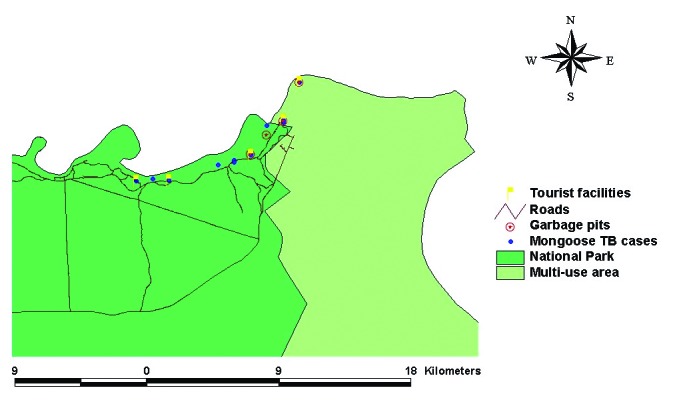
Locations of *Mycobacterium tuberculosis*-infected or suspected cases in banded mongooses, in relation to garbage pits, tourist facilities, and land use type, Chobe District, Botswana.

### Histopathologic and Bacteriologic Testing and Polymerase Chain Reaction Amplification

Various organs and tissues from seven banded mongooses and a suricate were fixed in 10% buffered Formol-saline, embedded in paraffin wax, cut into 4-µm sections, and stained with hematoxylin and eosin, as well as Kinyoun’s method for acid-fast bacteria (AFIP modification) (7).

Pooled organ specimens from one banded mongoose (No. 2075) and one suricate (No. VLF005) and liver samples from another banded mongoose (No. 2344) were homogenized, and equal parts were decontaminated with either 2% HCl or 4% NaOH. After neutralization, sediments were placed on two slants of Löwenstein-Jensen (LJ) medium containing 0.5% pyruvate and one slant containing glycerine. The cultures were incubated at 37°C and checked weekly for growth. Acid-fast culture isolates were subjected to polymerase chain reaction (PCR) specific for *M. tuberculosis-*complex organisms (8) and subcultured onto the same type of medium for identification by growth characteristics and standard biochemical tests. PCR-positive *Mycobacterium* isolates were subjected to a second PCR test to differentiate *M. bovis* and *M. tuberculosis* (9).

## Results

### Epidemiologic Features

In the suricates, the epizootic occurred from October 1998 to December 1999, after an unknown infected male joined a study group consisting of 5 adult suricates and 15 pups. One human case of TB was known to have occurred in the study area in the human population living near the suricate burrows. Suricates were not observed feeding in garbage pits; however, they were seen foraging around roads and investigating human sputum.

In the banded mongooses, the epizootic spread quickly in six troops living along the Chobe River Front. New clinical cases were identified from the initial outbreak site at an average rate of 0.109 km/day into Chobe National Park and the township at an average rate of 0.282 km/day. Although the last case was observed on September 15, monitoring continued until January 21, 2001; no new cases were identified. Garbage pits and a known human TB case were in close proximity to initial outbreak points (Figure). Banded mongooses were observed feeding regularly at these garbage pits and would therefore be exposed to human excretions and any infectious material from TB-infected humans.

### Clinical Signs and Gross Pathologic Findings

The first suricate noted to have clinical signs had enlarged cervical lymph nodes when it emigrated to the study site. The lymph nodes ruptured within a month and continued to discharge pus for 2 months (November–December 1998). By January 1999 the lesion had become a persistent nonhealing wound. The animal became progressively more debilitated and cachectic until he disappeared from the study site in April 1999. In June, clinical signs first appeared in other animals and then spread through the entire troop by December 1999. The signs included emaciation, weakness, and dyspnea with variable enlargement of the lymph nodes of the head, neck, and axilla. All troop members were euthanized, died, or disappeared and were presumed dead. Gross postmortem examination of one suricate showed abscesses filled with yellow fluid in the left parotid (3 cm in diameter), right axilla (1 cm), lung, liver, and mediastinal lymph nodes (5 mm), and pancreas (8 mm). The lungs were congested and edematous.

The mongoose troop size ranged from 8 to 35; in each troop, 2 to 4 animals had clinical signs. Affected animals were often found separated from the troop during periods of foraging. In some cases, although alert, they showed a pronounced lack of fear response to humans. Affected animals that were not euthanized disappeared within a few days after onset of illness and were presumed dead. Clinical signs included progressive cachexia, ataxia, and weakness. No other external abnormalities were noted. Necropsies of seven animals from five troops showed numerous miliary grayish white nodular masses (0.5 cm–2 cm in diameter) scattered over the liver and spleen surfaces, causing massive enlargement of the two organs; numerous grayish white infiltrative masses in the lung and kidney; enlargement of the mesenteric lymph nodes, which had necrotic gritty centers with chalky material; and occasional grayish white foci scattered throughout the length of the intestines.

### Histopathologic Findings

Granulomas of varying size, predominantly consisting of aggregated epithelioid macrophages, were found in most of the organs and tissues examined and were consistently present in the liver, spleen, lymph nodes, and lungs. Such granulomas were occasionally noted in the adrenal gland, kidney, myocardium, pancreas, epididymus, pleura, intestine, peritoneum, and skin. Lesions were absent from the brain, skeletal muscle, urinary bladder, and testis. The smaller granulomas consisted purely of macrophages, while large ones showed central necrosis and sometimes contained small aggregates of lymphocytes and plasma cells. Giant cells were rare, and no calcification was seen. Acid-fast rods, typical of *Mycobacterium* species, were noted in the cytoplasm of macrophages in all eight mongooses but varied in numbers from scarce to abundant.

### Bacteriologic and PCR Amplification Results

Acid-fast bacteria were detected on Ziehl-Neelsen–stained impression smears, and *Mycobacterium* species were isolated from both banded mongooses and the suricate specimens. For material from animal No. 2075, growth first appeared after 2 weeks on LJ slants with and without pyruvate. LJ-pyruvate cultures of the specimens from mongoose No. 2344 and suricate No. VLF005 yielded very few acid-fast colonies after a 5- to 6-week incubation period. Following PCR amplification, the *Mycobacterium* sp. isolated from mongoose No. 2344 and the suricate produced a 372-bp DNA product. Both isolates showed a 336-bp DNA product, characteristic for *M.* *tuberculosis* when amplified by the protocol of de Wit et al. (9). This method had been found useful in differentiating isolates of *M. bovis* and *M. tuberculosis* (A. Michel, unpub. data). The isolates produced positive results in both the niacin production and nitrate reduction tests, confirming them as *M. tuberculosis*. A subculture of an isolate from banded mongoose No. 2075 was classified as a fast-growing *Mycobacterium* species after it had shown growth at 27°C and 37°C at 7 and 5 days, respectively. No growth was observed at 45°C.

## Discussion

Because the lesions in the mongooses and the suricate were disseminated, the route of infection is not clear. However, an oral route of infection is suspected because the pulmonary lesions involved the interstitium and alveolar walls rather than the bronchioles, and mesenteric but not pulmonary lymph nodes were enlarged (banded mongoose). The behavioral pattern of both species would have facilitated exposure to human excretions in the environment and therefore to *M. tuberculosis* from any TB-infected humans.

Ecologic, environmental, and demographic factors influence the emergence of disease (10). TB incidence is increasing rapidly throughout the world with most cases in developing countries. In 1996, the Western Cape of South Africa had one of the highest incidences of human TB in the world (11). In Botswana, where most people are likely to have been infected with TB by adulthood (12), the TB infection rate in humans increased from 202 per 100,000 in 1989 (13) to 537 per 100,000 in 1999 (14). Concurrent HIV infection may shorten the time for TB infection to progress to overt disease, leading to increased severity of clinical signs and amount of Mycobacteria shed into the environment (15). In 1999, HIV sentinel surveillance in Botswana indicated that 36% of women receiving routine antenatal care were seropositive for HIV (16). In addition, concurrent helminthic infections may decrease the host immune response to TB, leading to reactivation of latent TB infections in humans and possibly increasing the level of TB in a community (17). Research is urgently required to better understand the epidemiology of *M. tuberculosis* in free-ranging wildlife and their potential to maintain infection in the absence of human reservoirs. Another factor influencing disease emergence is the dramatic increase in travel. In 1999, >89,000 visitors were recorded in Chobe National Park (18). Changes in the health, mobility, and number of humans in the vicinity of wildlife may have led to an increased level of *M. tuberculosis* being shed into the environment, resulting in this spillover of infection into a wildlife population.

Expansion of ecotourism, changes in land-use practices, and escalating competition for resources has increased contact between free-ranging wildlife and humans. Tremendous attention has been given to the zoonotic potential of emerging diseases in wildlife populations and the threat they present to human health. Little attention, however, has been given to the reverse: the disease threat humans present to wildlife. A number of reports have suggested links between pathogen occurrence in wildlife populations and human exposure, but the diagnoses were not confirmed and proof of transmission was lacking (19). In other cases involving macroparasites, the pathogens have been found in a number of domestic or free-ranging wildlife hosts, complicating transmission routes and links to human reservoirs (20,21). This report, however, represents the first clearly documented case of a primary human pathogen infecting free-ranging wildlife. The report underscores the need to heighten awareness of humans as a potential reservoir of disease for wildlife and the role humans may play in the emergence of infectious disease in wildlife populations. This understanding will be essential for developing effective programs for public and wildlife health.

Ecotourism brings large numbers of people to wildlife areas and provides both important economic benefits and an instrument for the conservation of biodiversity. However, susceptible wildlife populations may be negatively affected by the increased exposure to humans and their pathogens. A better understanding of the dynamics of disease transmission between humans and wildlife is critical, and mechanisms must be identified that limit wildlife exposure to human pathogens. Attention to this area of wildlife management is essential to the long-term conservation and sustainable use of wildlife resources.
